# Pain self‐management intervention supports successful attainment of self‐selected rehabilitation goals—secondary analysis of a randomized controlled trial

**DOI:** 10.1111/hex.13469

**Published:** 2022-03-14

**Authors:** Catharina Gustavsson, Lena von Koch

**Affiliations:** ^1^ Center for Clinical Research Dalarna Uppsala University Falun Sweden; ^2^ Department of Public Health and Caring Science Uppsala University Uppsala Sweden; ^3^ School of Health and Welfare Dalarna University Falun Sweden; ^4^ Department of Neurobiology, Care Science and Society Karolinska Institutet Stockholm Sweden; ^5^ Theme Neuro Karolinska University Hospital Stockholm Sweden

**Keywords:** activities of daily living, disability, persistent pain, person‐centred care, self‐efficacy, self‐management

## Abstract

**Objectives:**

(i) Describe patients' self‐selected activity‐related rehabilitation goals, and (ii) compare attainment of these rehabilitation goals among people with persistent tension‐type neck pain receiving a group‐based pain and stress self‐management intervention (PASS) or individual physiotherapy (IPT).

**Methods:**

Before intervention and random allocation to PASS or IPT, 156 people (PASS *n* = 77, IPT *n* = 79), listed three self‐selected activity‐related rehabilitation goals by use of the Patient Goal Priority Questionnaire (PGPQ). For each activity goal, participants rated limitations in activity performance, self‐efficacy and fear of activity performance, readiness to change to improve performance, and expectations of future activity performance. At follow‐ups (10 weeks, 20 weeks, 1 year and 2 years after inclusion), participants also responded to a question on changes made to improve activity performance. Mann–Whitney *U* test was used to evaluate between‐group differences.

**Results:**

There were between‐group differences in favour of PASS in the attainment of self‐selected rehabilitation goals with regard to activity limitations and satisfaction with activity performance at all follow‐ups.

**Conclusions:**

PASS was more successful than IPT for the attainment of self‐selected rehabilitation goals, improvements in activity limitations and satisfaction with activity performance as measured by PGPQ. The PASS programme emphasized the importance of applying active pain‐ and stress‐coping techniques in personal ‘risk situations’ for pain flare‐ups, which appear to support people with persistent tension‐type neck pain to make changes in their lives to improve activity performance.

**Patient or Public Contribution:**

Patient engagement in rehabilitation by self‐selected goals was investigated, but patients were not involved in the design or conduct of the study.

## 1 INTRODUCTION

The biopsychosocial perspective on pain highlights the significance of applying cognitive‐behavioural principles in pain rehabilitation.^1^, ^2^, ^3^ Rehabilitation strategies that address cognitive and behavioural factors involved in the maintenance of musculoskeletal pain by promoting active pain‐coping skills and self‐management have shown beneficial effects on pain‐related disability.^4^, ^5^, ^6^ The rationale for self‐management interventions is to induce health behaviour assumed to yield sustainable long‐term favourable effects on disability.^7^, ^8^

Person‐centred care and the patient's active involvement in rehabilitation have gained increased attention.^9^, ^10^, ^11^, ^12^ An important part of a person‐centred approach is the identification and evaluation of patient‐specific rehabilitation goals.^13^, ^14^ Clinical tools can support the goal‐setting process: assist in drawing out and setting priority to the patient's valued treatment goals, and monitor the treatment process and goal achievements.^15^ Patient‐specific instruments are useful for bringing forward and acknowledging the individual's own valued activities, such as activities that are perceived important for the person's participation in everyday life and for actively involving the patient in the rehabilitation process.^16^, ^17^, ^18^ The patient's active involvement in rehabilitation goal‐setting has been suggested to increase motivation, active engagement and satisfaction with rehabilitation interventions.^19^ Studies have found a positive association between patient involvement in goal‐setting and rehabilitation outcome.^20^, ^21^ Despite support for the use of patient‐specific instruments for goal‐setting, they are used to a very small extent in clinical rehabilitation.^18^, ^22^, ^23^

The Patient Goal Priority Questionnaire (PGPQ) has been described as a clinical tool to assist the patient in identifying and prioritizing activity‐related goals in pain rehabilitation, likewise to assist shared decision‐making and patient engagement in the evaluation of rehabilitation progress and outcome.^24^ The activity goals rated by PGPQ are self‐selected by the patient and thus person‐specific as opposed to generic. Each prioritized activity goal is followed by a rating of generic aspects relating to activity performance; that is, limitations in activity performance, as well as satisfaction, self‐efficacy and fear of activity performance, readiness to change to improve performance and expectations of future activity performance. One study has examined test‐retest reliability of the self‐report scales in PGPQ in a sample of people having persistent pain by calculating intra‐class correlation coefficients (ICCs) and the reported ICCs ranged from .35 to .81.^25^ The PGPQ has been used in pain rehabilitation research for the identification of patients' treatment goals^26^ and for the assessment of clinically important changes related to activity performance and goal achievements.^27^, ^28^ One study examined the concurrent validity of the PGPQ to a generic measure of disability, the Pain Disability Index (PDI), in a sample of people having persistent pain. The PGPQ was negatively and moderately correlated with the PDI, indicating that the patients' perceptions of behavioural performance in prioritized activity goals as measured by PGPQ were moderately correlated to a generic measure of disability.^24^

In a randomized controlled trial (RCT) of a multicomponent pain and stress self‐management group intervention (PASS), the PGPQ was included in a comprehensive self‐assessment questionnaire used for evaluation of intervention effects. Previous publications have reported results on posttreatment effects in the primary outcome: pain control, self‐efficacy for activities of daily living (ADL) and disability^29^ and on long‐term effects at 2^30^, ^31^ and 9 years.^32^ The results on primary outcomes have shown that the PASS had a better effect on pain control, pain‐related self‐efficacy for ADL, disability and pain catastrophizing than a control treatment: individual physiotherapy (IPT), for patients with persistent tension‐type neck pain, in both short‐term and long‐term.^29^, ^30^ Also, treatment gains in self‐efficacy for activity performance was an important predictor for favourable long‐term outcomes on pain‐related disability.^31^ However, the outcome on the participants' attainment of self‐selected rehabilitation goals collected by PGPQ before the intervention and at follow‐ups (at 10 weeks, 20 weeks, 1 year and 2 years after inclusion) has not yet been reported.

Hence, the objective of this study was to (i) describe patients' self‐selected activity‐related rehabilitation goals, and (ii) compare attainment of these rehabilitation goals as measured by PGPQ among people with persistent tension‐type neck pain participating in a two‐armed RCT receiving either PASS intervention or IPT.

## 2 MATERIALS AND METHODS

### 2.1 Study design

This study is a secondary analysis of data from a two‐armed pragmatic^33^ RCT^34^ evaluating between‐group differences and within‐group changes over time with five time‐points of data collection, on activity performance in self‐selected rehabilitation goals.

### 2.2 Participants and procedures

Detailed descriptions of participants and procedures have been reported in previous publications.^29^, ^30^, ^31^ In brief, people with neck pain seeking physiotherapy treatment at nine primary healthcare (PHC) centres in eight towns in Sweden were consecutively recruited from September 2004 to April 2006. They were considered eligible if they were aged 18–65 years and had tension‐type neck pain of persistent duration; that is, more than 3 months. Reasons for exclusion were: insufficient fluency in Swedish, medical history of psychotic disorder, pregnancy, ongoing treatment for neck pain or possible depression indicated by a score of ≥11 points on the depression subscale of the Hospital Anxiety and Depression Scale (HADS‐D).^35^ An *a‐priori* power calculation based on data from a pilot study,^36^ estimated that a sample size of 55 per group would be sufficient to detect a 10% between‐group difference on the primary outcomes variables: ‘pain control’ and ‘self‐efficacy for performing activities in spite of pain’. No power calculation was undertaken regarding outcome on the PGPQ. The number of participants available for analyses of PGPQ was considered acceptable for analyses but the capacity to ensure power to detect important between‐group differences was not calculated a priori. Before enrolment to the study, the participants provided informed consent to participate. The study was approved by the Ethical Review Board of Uppsala University (Ups02‐088). After completing the baseline self‐assessment questionnaire, the participants were randomly allocated to receive either the intervention PASS or the control condition IPT. Allocation was stratified by the PHC centre. PASS and IPT were delivered at the PHC centres by experienced physiotherapists. The physiotherapists delivering PASS and IPT did not have access to the content of the questionnaire and were unaware of participants' responses to the PGPQ. Follow‐ups were conducted 10 weeks, 20 weeks, 1 year and 2 years after inclusion by use of self‐assessment questionnaires, which were mailed to the participants. In the present study, responses to the PGPQ at baseline and follow‐ups were analysed.

### 2.3 PASS and IPT interventions

PASS was a multicomponent PASS consisting of seven weekly group sessions of 1.5 h each, and an additional booster session at 20 weeks after the initial session. Each session consisted of applied relaxation,^37^ body‐awareness exercises^38^ and interactive lectures, emphasizing two‐way communication and group discussions concerning issues related to pain self‐management.^29^ The rationale was to teach the patient active pain‐ and stress‐coping skills by identifying personal ‘risk situations’ in everyday life (i.e., activities, movements or thoughts believed to cause pain or stress) and applying techniques in these situations to manage physical arousal, and thereby prevent the pain from starting or increasing. The PASS participants attended an average of seven (range: 4–8) group sessions.^29^

IPT was individually administered physiotherapy sessions in accordance with current practice at the PHC centres and was not standardized; that is, type of treatment, frequency of visits and duration of contact were left to the judgement and agreement between the physiotherapist and the patient. The sessions involved several treatment modalities: spinal mobilization techniques and massage, acupuncture, transcutaneous electric nerve stimulation and individually tailored exercise programmes. The IPT participants received an average of eleven (range: 1–52) individual sessions.^29^

### 2.4 Data collection

The PGPQ was used to collect data concerning patients' priorities of rehabilitation goals.^24^ The participants selected three activity‐related rehabilitation goals; that is, activities they were unable or had difficulties performing due to pain, and they wanted to improve through rehabilitation. They ranked the relative importance of the activities from 1 to 3, with 1 representing the most important activity (referred to as Activity 1, 2 and 3 in this paper). For each activity, the participants rated the following aspects:

1. *current limitations in activity performance* on an 11‐point numeric rating scale (NRS) (higher scores indicating more severe activity limitations);
2. *frequency of activity performance* during the past week on a 5‐point ordinal scale (0 = *never*, 1 = *once*, 2 = *twice*, 3 = *3 to 5 times*, 4 = *more than 5 times*);
3. *satisfaction with the current level of activity performance* on 11‐point NRS (higher scores indicating higher satisfaction);
4. *self‐efficacy for activity performance despite pain* on 11‐point NRS (higher scores indicating higher self‐efficacy);
5. *fear of activity performance* on 11‐point NRS (higher scores indicating more fear);
6. readiness to change (adopt new behaviours) to improve activity performance on an 11‐point NRS (higher scores indicating higher readiness); and
7. *expectations of future activity performance* as a result of rehabilitation on an 11‐point NRS (higher scores indicating expectations of more limitations in activity performance).
8. At follow‐ups, aspects 6 and 7 were omitted, and an additional rating was included concerning the *amount of changes made to improve activity performance*, scored on a 4‐point ordinal scale (0 = *none*, 1 = *a few changes*, 2 = *some changes*, 3 = *many changes*). At the follow‐ups, each individual's original self‐selected rehabilitation goals as stated in the baseline questionnaire were filled in before the questionnaire was sent to the participant.

### 2.5 Data management and analyses

The PDI^39^, ^40^ was used to categorize the patients' self‐selected rehabilitation goals listed in the PGPQ according to activity domains. The PDI has been used in other studies to categorize the patient‐specific activities in the PGPQ into generic activity domains.^24^, ^25^ The PDI is a generic instrument designed to assess pain‐related interference with activities and role functioning in seven domains: (1) family and home responsibilities; (2) recreation and hobbies; (3) social activity; (4) occupation and education; (5) sexual behaviour; (6) self‐care and (7) life‐support activities. The two authors performed the categorization of activities to PDI domains using qualitative analysis. Both authors independently examined the data set and categorized each activity goal into one of the seven PDI domains. Then, the authors compared the categorizations. There was almost a complete inter‐rater agreement. The few disagreements were discussed until consensus was reached and the categorization was revised. Activity goals that were interpreted as covering more than one PDI domain were allotted to a separate category. An additional category was included and labelled ‘general functional ability’, comprising general activities not tied to any specific task or situation, for example, sitting, standing, walking and handling objects. In addition, priorities that were not considered activity‐related rehabilitation goals, such as ‘get well’, ‘get less pain’ and ‘feel less tired’, were categorized as ‘Not activity‐related goals’.

Mann–Whitney *U* test^41^ was used to evaluate differences between groups at baseline and follow‐ups (10 weeks, 20 weeks, 1 year and 2 years), in measures of aspects relating to activity performance as rated by PGPQ. Data were analysed *per protocol*; that is, based on participants with available data at each follow‐up and according to group allocation at baseline.^42^ To adjust for multiple testing; that is, five time points of follow‐ups and for all three goal activities, a *p*‐value less than .01 was accepted as statistically significant.^41^ Analyses were conducted using IBM SPSS Statistics 21 for Windows.^43^

## 3 RESULTS

**Figure 1:**
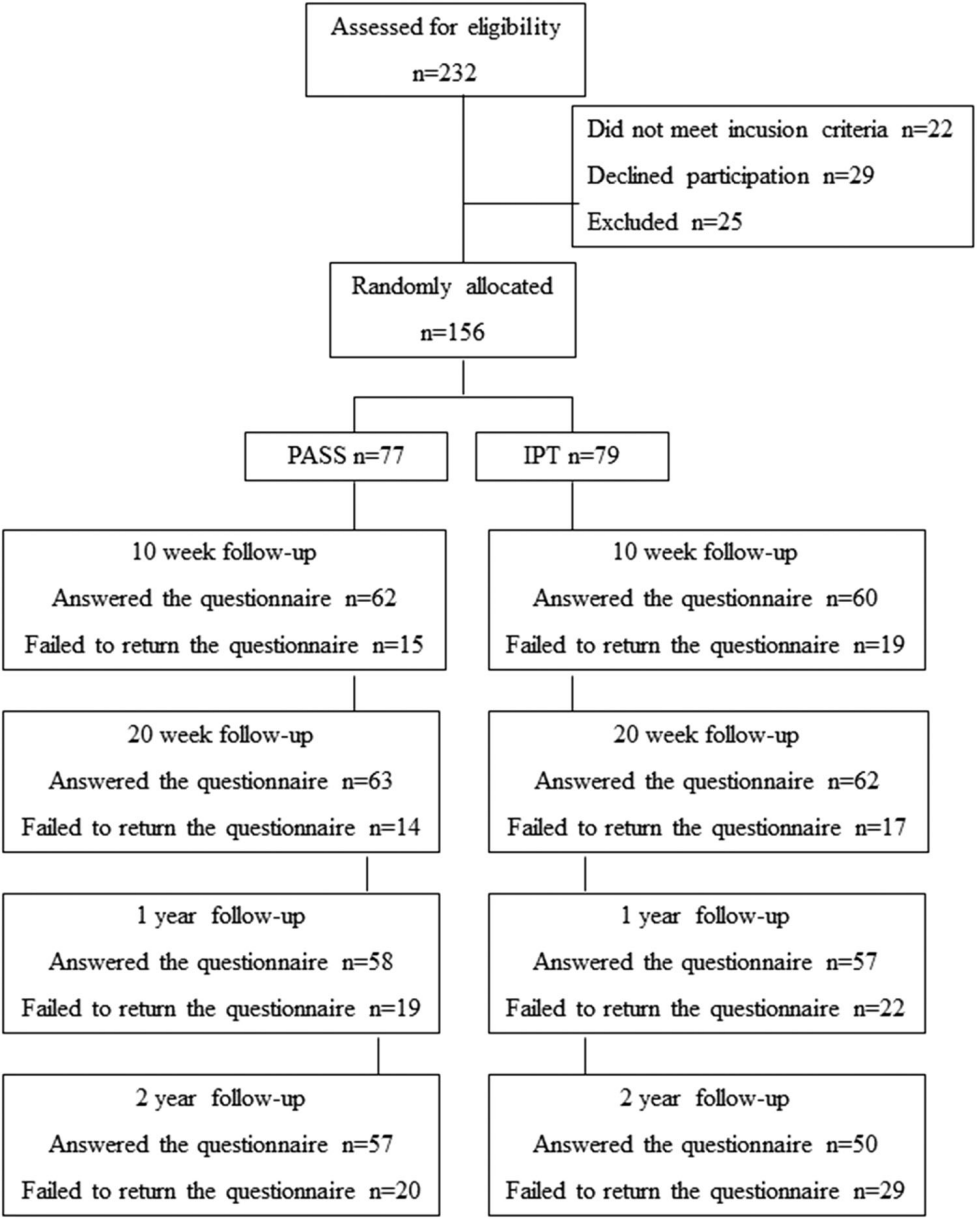
Flowchart of participants in the randomized controlled trial and numbers (*n*) responding to follow‐ups. IPT, individual physiotherapy control group; PASS, pain and stress self‐management intervention group

Baseline characteristics of the 156 participants who were originally included in the RCT, PASS *n* = 77; IPT *n* = 79, are displayed in Table 1. At follow‐ups, 20%–37% of participants failed to respond to the self‐assessment questionnaires. Figure 1 provides a flowchart illustrating participation in the study over the follow‐ups.

**Table 1:** Baseline characteristics for participants in pain and stress self‐management (PASS) group and in individual physiotherapy (IPT) group

	PASS‐group (*n* = 77)	IPT‐group (*n* = 79)
Female/male *n* (%)	69 (90)/8 (10)	70 (89)/9 (11)
Age mean (SD) range	45.7 (11.5) 19–65	45.7 (11.6) 20–63
Pain intensity		
Present (0–10) mean (SD)	5.5 (2)	5.9 (2)
Average (0–10) mean (SD)	6 (1.8)	6.4 (2)
Worst/maximum (0–10) mean (SD)	8.4 (1.4)	8.5 (1.2)
Duration of neck pain		
3–6 months *n* (%)	7 (9)	11 (14)
7–12 months *n* (%)	4 (5)	9 (11)
1–2 years *n* (%)	12 (16)	12 (15)
More than 2 years *n* (%)	54 (70)	47 (60)
NDI: Neck Disability Index (0–100) mean (SD)	30.8 (10.7)	35.4 (14)
SES: Self‐Efficacy Scale (0–200) mean (SD)	136.7 (39.8)	128.3 (43.5)
CSQ Pain control (0–6) mean (SD)	3.3 (1.1)	3.1 (1.2)
CSQ Ability to reduce pain (0–6) mean (SD)	2.9 (1)	2.9 (0.9)
CSQ Catastrophizing (0–36) mean (SD)	11.3 (7.4)	11.8 (7.1)
HADS‐Depression Subscale (0–21) mean (SD)	4.3 (3.1)	4.9 (8.9)
HADS‐Anxiety Subscale (0–21) mean (SD)	8.2 (4.1)	8.1 (3.9)

Abbreviations: CSQ, Coping Strategies Questionnaire; HADS, Hospital Anxiety and Depression Scale; SD, standard deviation.

Table 2 displays the categorization of patients' self‐selected activity‐related rehabilitation goals according to the activity domains in PDI. Activities related to ‘Occupation and Education’ were the most frequent in Activity 1, constituting 32% of all listed rehabilitation goals. Activities related to ‘Recreation and Hobbies’ were the most frequent rehabilitation goals in Activity 2 (46%) and Activity 3 (41.5%). Among the activity‐related goals deemed covering more than one activity domain, the most frequent combination was ‘Occupation and Education’ and ‘Recreation and Hobbies’. An example of such a combined goal is ‘Sitting upright in front of a computer at work or a TV screen to watch a TV programme for more than 15 min’. Very few the participants' rehabilitation goals were categorized as not activity‐related (4.4%).

**Table 2:** Results of categorization of participants' prioritized rehabilitation goals in the PGPQ form at baseline by life‐functioning domains in Pain Disability Index

**Goal category**	Activity 1	Activity 2	Activity 3
	PASS (*n* = 77), IPT (*n* = 79)	PASS (*n* = 75), IPT (*n* = 78)	PASS (*n* = 70), IPT (*n* = 72)
Family and home responsibilities			
PASS, number (%)	10 (13.0)	12 (16.0)	16 (22.9)
IPT, number (%)	11 (13.9)	13 (16.7)	13 (18.1)
Recreation and hobbies			
PASS, number (%)	16 (20.8)	36 (48.0)	28 (40.0)
IPT, number (%)	20 (25.3)	34 (43.6)	31 (43.1)
Social activity			
PASS, number (%)	1 (1.3)	1 (1.3)	0 (0.0)
IPT, number (%)	0 (0.0)	0 (0.0)	2 (2.8)
Occupation and education			
PASS, number (%)	29 (37.7)	10 (13.3)	7 (10.0)
IPT, number (%)	21 (26.6)	11 (14.1)	3 (4.2)
Self‐care			
PASS, number (%)	1 (1.3)	2 (2.7)	4 (5.7)
IPT, number (%)	3 (3.8)	1 (1.3)	8 (11.1)
Life‐support activities			
PASS, number (%)	5 (6.5)	0 (0.0)	1 (1.4)
IPT, number (%)	1 (1.3)	2 (2.6)	3 (4.2)
Activity goal covering more than one activity domain			
PASS, number (%)	7 (9.1)	4 (5.3)	4 (5.7)
IPT, number (%)	11 (13.9)	7 (9.0)	3 (4.2)
General functional ability			
PASS, number (%)	7 (9.1)	6 (8.0)	5 (7.1)
IPT, number (%)	9 (11.4)	7 (9.0)	5 (6.9)
Not activity‐related goal			
PASS, number (%)	1 (1.3)	4 (5.3)	5 (7.1)
IPT, number (%)	3 (3.8)	3 (3.8)	4 (5.6)

Abbreviations: IPT, individual physiotherapy control group; PASS, pain and stress self‐management intervention group; PGPQ, Patient Goal Priority Questionnaire.

Table 3 presents between‐group comparisons for ratings of aspects of activity performance for the participants' self‐selected rehabilitation goals as assessed by PGPQ. The between‐group differences at follow‐ups were all in favour of the PASS. Concerning ‘limitations in activity performance’ and ‘satisfaction with activity performance’, there were between‐group differences in favour of PASS at all follow‐ups. Regarding ‘Fear of activity performance’, there was a between‐group difference only at 10‐week follow‐up and only for Activity 1 (*p* = .010), also in favour of PASS.

**Table 3:** Responses to PGPQ items for PASS and IPT at baseline and follow‐ups and comparison of differences between groups based on participants with available data at follow‐ups

							Baseline						10‐week follow‐up					
							Activity 1		Activity 2		Activity 3		Activity 1		Activity 2		Activity 3	
*Item in PGPQ (response option values)*							PASS (*n* = 77), IPT (*n* = 79)^a^		PASS (*n* = 75), IPT (*n* = 77)^a^		PASS (*n *= 70), IPT (*n* = 71)^a^		PASS (*n* = 59), IPT (*n* = 55)^a^		PASS (*n* = 58), IPT (*n* = 56)^a^		PASS (*n* = 53), IPT (*n* = 52)^a^	
*Treatment group*							Md (IQR)	*p* Value^b^	Md (IQR)	*p* Value^b^	Md (IQR)	*p* Value^b^	Md (IQR)	*p* Value^b^	Md (IQR)	*p* Value^b^	Md (IQR)	*p* Value^b^
Item 1: Current limitations in activity performance (0–10, higher score more limitations)								.086		.155		.500		**.003**		**.001**		.134
PASS‐group							6 (4–8)		7 (5–9)		7 (5–10)		4 (2–6)		4 (2–6)		5 (1.5–6.5)	
IPT‐group							7 (5–9)		8 (5–9)		7 (6–10)		5 (4–9)		7 (5–9.75)		5 (3–8.25)	
Item 2: Frequency of activity performance past week (0–4, higher score more frequent)								.551		.647		.907		.221		**.004**		.519
PASS‐group							3 (1–4)		1.5 (1–3)		2 (0–3)		3 (1–4)		3 (2–3.25)		2 (1–3)	
IPT‐group							3 (1–4)		2 (0–3)		1 (0–3)		3 (1–4)		2 (0–3)		2 (0.25–3)	
Item 3: Satisfaction with activity performance (0–10, higher score higher satisfaction)								.257		.992		.051		**.005**		**<.0001**		.026
PASS‐group							3 (2–5)		3 (0–4)		2 (0–4)		6 (3–9)		6.5 (3–8)		6 (3–8)	
IPT‐group							3 (0–5)		3 (0–5)		3 (1–5)		4 (2–7)		4 (0–5)		4 (1–56)	
Item 4: Self‐efficacy for activity performance (0–10, higher score higher self‐efficacy)								.349		.738		.995		.023		.051		.131
PASS‐group							4 (2–6.5)		2 (1–5)		2 (1–5)		5 (3–8)		4 (2.75–8)		5 (2.5–7.5)	
IPT‐group							3 (1–5)		3 (1–5)		3 (1–5)		3.5 (1–5)		3 (1–5)		4 (2–6)	
Item 5: Fear of activity performance (0–10, higher score more fear)								.608		.833		.962		**.010**		.126		.164
PASS‐group							5 (1.5–7)		5 (2–8)		5 (2–8)		2 (0–6)		3 (0–6		3 (0–5.5)	
IPT‐group							5 (2–7)		6 (3–8)		5 (2–8)		4 (1–7)		4 (1–8)		3.5 (2–6)	
Item 6: Readiness to change to improve activity (0–10, higher score higher readiness)								.411		.080		.137						
PASS‐group							10 (9–10)		10 (9–10)		10 (9–10)		–		–		–	
IPT‐group							10 (8–10)		10 (8–10)		10 (8–10)		–		–		–	
Item 7: Expectations of future activity performance as a result of treatment (0–10, higher score expecting more limitations)							.076		.084		.246							
PASS‐group							1 (1–2.75)		2 (0–3)		2 (0–3)		–		–		–	
IPT‐group							2 (1–4)		2 (1–5)		2 (1–4)		–		–		–	
Item 8 follow‐up: Amount of changes made by the patient to improve activity performance (0–3, higher score more changes made)														.023		**.002**		**.003**
PASS‐group							–		–		–		1 (1–1)		1 (1–1)		1 (1–1.75)	
IPT‐group							–		–		–		1 (0–1)		1 (0–1)		1 (0–1)	

	**20‐week follow‐up**						**1‐year follow‐up**						**2‐year follow‐up**					
	**Activity 1**		**Activity 2**		**Activity 3**		**Activity 1**		**Activity 2**		**Activity 3**		**Activity 1**		**Activity 2**		**Activity 3**	
*Item in PGPQ*	PASS (*n* = 63), IPT (*n* = 62)^a^		PASS (n = 56), IPT (*n* = 59)^a^		PASS (*n* = 55), IPT (*n* = 55)^a^		PASS (*n* = 56), IPT (*n* = 57)^a^		PASS (*n* = 55), IPT (*n* = 55)^a^		PASS (*n* = 52), IPT (*n* = 52)^a^		PASS (*n* = 55), IPT (*n* = 49)^a^		PASS (*n* = 54), IPT (*n* = 49)^a^		PASS (*n* = 53), IPT (*n* = 45)^a^	
*Continued*	Md (IQR)	*p* Value^b^	Md (IQR)	*p* Value^b^	Md (IQR)	*p* Value^b^	Md (IQR)	*p* Value^b^	Md (IQR)	*p* Value^b^	Md (IQR)	*p* Value^b^	Md (IQR)	*p* Value^b^	Md (IQR)	*p* Value^b^	Md (IQR)	*p* Value^b^
Item 1		.021		**.007**		.160		**.001**		**.001**		.375		**.008**		**.011**		.022
PASS‐group	3 (1–7)		3 (2–6.75)		4 (2–6)		3 (1–5)		3 (1–6)		4 (2–7.75)		3 (1–6)		2.5 (0–8)		2 (1–5.5)	
IPT‐group	5 (3–8)		6 (3–8)		5 (2–8)		5 (3.5–7)		6 (3–8)		5 (2–7)		5 (2.5–8)		6 (4–8)		5 (2–8)	
Item 2		.886		.194		.695		.807		.592		.639		.962		.580		.396
PASS‐group	3 (2–4)		3 (1–4)		2 (1–3)		3 (2–4)		3 (1–4)		2 (1–4)		3 (1–4)		2 (1–3.25)		2 (1–4)	
IPT‐group	3 (1–4)		2 (0–3)		2 (1–3)		3 (1.25–4)		2 (1–4)		2 (1–3)		3 (1.5–4)		3 (1–3)		2 (0.5–3)	
Item 3		**.011**		**.014**		.303		**.003**		**.006**		.416		**.003**		**.013**		.034
PASS‐group	7 (4–9)		7 (4.25–9)		5 (3–8)		7.5 (4–9.75)		7 (3–9)		5 (2–8)		8 (5–9.25)		8 (4–10)		7 (4–9)	
IPT‐group	5 (2.5–8)		5 (2.75–8)		4 (2–8)		4.5 (2–7)		4 (2–7)		5 (2–7.75)		4 (2–8)		5 (2–7)		4 (2–7)	
Item 4		.136		.065		.101		.046		.323		.066		.164		.034		.082
PASS‐group	4 (3–8)		5 (3–8)		5 (2.25–8)		5 (3–8)		5 (2.25–8)		5 (2.5–8)		7 (3–8)		6 (3.5–9)		6 (3–9)	
IPT‐group	4 (2–7)		4 (2–7)		4 (2–6)		4 (2–7)		4 (2–7)		4 (2–6)		5 (2–8)		5 (2–7)		5 (2–7.5)	
Item 5		.649		.561		.647		.134		.616		1.000		.329		.596		.603
PASS‐group	3 (0–5)		3 (0.25–6)		3 (0–6)		2 (0–5)		2.5 (0–5)		4 (0.5–6)		1 (0–5)		2 (0–5)		1 (0–5)	
IPT‐group	3 (1–5)		3 (1–7)		3 (1–6)		3.5 (1–5.75)		3 (1–6)		3 (1‐5.5)		3 (0–5)		2 (0–5)		2 (0–6)	
Item 8		.223		.146		.531		.606		.168		.899		.077		.496		.929
PASS‐group	1 (1–1)		1 (0–1)		1 (0–1)		1 (0–2)		1 (0–1)		1 (0–1)		1 (0–2)		1 (0–2)		0 (0–1)	
IPT‐group	1 (0–1.25)		1 (0–1)		1 (0–1)		1 (0–1)		1 (0–1)		0 (0–1)		1 (0–2)		1 (0–1)		0 (0–1.5)	

Note: Bold values indicate *p*‐value ≤ 0.01.

Abbreviations: IPT, individual physiotherapy; IQR, interquartile range; Md, median; PASS, pain and stress self‐management intervention; PGPQ, Patient Goal Priority Questionnaire.

^a^ Number of participants providing data on activity goals at each follow‐up.

^b^ Significance test by Mann–Whitney *U* test. Bold values indicate a *p*‐value ≤ 0.01.

Concerning ‘Amount of changes made by the patient to improve activity performance’, there were between‐group differences in favour of PASS at 10‐week follow‐up; that is, immediately after completing the group programme for Activity 2 (*p* = .002) and Activity 3 *p* = .003) but no between‐group differences at any other follow‐up.

## 4 DISCUSSION

This study showed that improvements in activity limitations and satisfaction with activity performance of self‐selected prioritized rehabilitation goals were greater in the PASS group as compared to the IPT group at intervention follow‐ups. This suggests that the PASS intervention was more successful in affecting activity limitations and satisfaction with activity performance than the IPT. The difference between groups was sustained over time up to 2 years postintervention. Also, at 10‐week follow‐up, immediately after completing the intervention, the PASS group reported having done more changes in their life to improve activity performance than the IPT group. The PASS programme emphasized the importance of applying active pain‐ and stress‐coping techniques in personal ‘risk situations’ in everyday life to manage physical arousal and thereby prevent the pain from increasing. Thus, we find it plausible that the PASS programme accounted for the changes made by the participants to their everyday life.

In previous publications from this RCT, we have reported favourable effects of PASS on pain control, pain‐related self‐efficacy for ADL and disability as measured by generic instruments.^29^, ^30^, ^31^ In this study, we aimed to explore effects on the self‐selected person‐specific activity‐related outcomes. The importance of the self‐selected person‐specific goal‐setting within physiotherapy has been raised as a means to identify goals that are perceived as meaningful and valued by the participant and worth investing time and effort to achieve.^19^ Indeed, it is plausible that the self‐selected activity goals in PGPQ enhanced motivation to invest own actions for change and engage in rehabilitation. However, the results of this study imply that the person‐specific goal‐setting before the intervention by use of PGPQ had less effect on the outcome of satisfaction with activity performance in prioritized goals in the IPT group as compared to the PASS group.

Although person‐specific goal‐setting instruments are incontrovertibly valuable in clinical physiotherapy practice, they may be difficult to use in research for evaluating patients' goal attainment. Person‐specific goals impose difficulties in making comparisons in‐between individuals. In this study, the participants expressed very different types of activities as prioritized rehabilitation goals. Thus, the question in PGPQ relating to the overall frequency of activity performance was not necessarily comparable in‐between individuals nor groups. For example, activities relating to self‐care or life‐supporting activities are typically performed several times a day, while activities relating to social activities probably are performed only once a day or a few times per week. However, the questions in PGPQ about activity limitations and satisfaction with activity performance, self‐efficacy for, fear of and expectations of future activity performance and amount of changes made in everyday life, are related to generic concepts and thus possible to compare in‐between individuals and groups. We suggest that a major advantage of the PGPQ, as compared to some other person‐specific goal‐setting instruments, is the inclusion of rating of important psychosocial factors; that is, self‐efficacy, fear and outcome expectations, which are known to impede activity performance in pain populations^44^ and affect rehabilitation success.^31^, ^45^ The patient‐specific functional scale (PSFS)^46^ is commonly used in physiotherapy practice and rehabilitation research.^47^ In PSFS, the patient is asked to provide three activities and rate their current ability to perform the activity, but there is no rating of relating generic psychosocial factors, which hamper comparison between individuals or aggregating data at a group level. The Canadian Occupational Performance Measure (COPM)^48^ is commonly used in occupational therapy practice and in rehabilitation research.^49^ The COPM is conducted by an interview. The patients indicate their problems in areas of self‐care, productivity and leisure. For each problem, the patient rates importance, performance and satisfaction with current performance using a scale from 1 to 10. Thus, by rating ‘satisfaction with activity performance’, the COPM includes a generic measure that allows for comparison between individuals, even when individuals prioritize different activity goals. However, the COPM does not include ratings of psychosocial factors known to be associated with activity performance and rehabilitation success in pain populations.

The PDI was used to categorize the participants' self‐selected rehabilitation goals into seven everyday life activity domains. The rehabilitation goals given the highest priority (Activity 1) were categorized to work‐related and education activities. Almost half of the goals prioritized as second and third (Activity 2 and 3), were categorized as recreational activities. Several of the rehabilitation goals covered more than one activity domain in PDI and also in such cases, the most common combination was work‐related/education and recreational activities. In an increasingly digitalized world, ‘sitting in front of a computer’ is needed in all areas of life, both working life, education and recreational activities. Åsenlöf et al.,^24^ found a similar distribution where the rehabilitation goals given the highest priority (Activity 1) most commonly belonged to the work and education domain. However, the goals prioritized as second and third (Activity 2 and 3), in that study most commonly were categorized to family and home responsibilities and to recreational activities as compared to the present study in which recreational activities were most common.

The written instruction in PGPQ to the participant was to formulate a specific activity‐related rehabilitation goal. Almost all participants managed to do so. Only very few participants' rehabilitation goals were categorized as not activity‐related (4.4%). A study^22^ that investigated the goal‐setting process in physiotherapy practice found that physiotherapists admitted that they were used to focussing on problems at the ICF‐level of body functions, instead of activities and participation. These physiotherapists used a goal‐setting instrument with limited patient involvement and without subsequently integrating the goal activities in the treatment process; they perceived goal setting as difficult and found themselves not fully prepared for involving the patients in this task.

A strength of this study is the follow‐up of the participants at 10 weeks, 20 weeks, 1 year and 2 years. This allowed for investigation of changes over time and sustainability of changes. It is a strength of the study that the participants prioritized their rehabilitation goals and rated activity performance before randomization to treatment group. Another strength is that all participants (both PASS and IPT participants) received the same information and instructions before goal setting using PGPQ and that the goal‐setting by PGPQ was not part of either rehabilitation programme. It cannot be ruled out that the patients in either group told their physiotherapists about their rehabilitation goals. If so, it is not known how such goals were handled by the physiotherapists.

The present study is a secondary analysis of an RCT conducted and completed more than 10 years ago, which may be considered a limitation. The main publication,^29^ outlining results on posttreatment effects in primary outcome variables, was published in 2010. The IPT provided in the study was equivalent to a regular practice of physiotherapy in PHC at the time of data collection. Since contemporary IPT for people with persistent tension‐type neck pain is delivered in a similar way today as when the RCT was undertaken, the results are still valid for current clinical practice. Moreover, as the aim of the present study was to compare goal attainment in goals set by the patients themselves, we believe that the research question addressed is of great interest and importance for the ongoing knowledge development of person‐centred rehabilitation. Self‐selected rehabilitation goals were to a larger extent attained and maintained in the group receiving the self‐management programme (PASS) than those receiving IPT. IPT may have included instructions for self‐management yet it did not lead to similar goal attainment of the patients' self‐selected goals.

It is a limitation that 20%–37% of participants failed to respond to follow‐up questionnaires despite reminders. In addition, the power calculation was based on the primary outcome variables in the RCT, not on the outcome of PGPQ. Still, the number of participants available for analyses of PGPQ was considered acceptable for analyses but limited the capacity to ensure power to detect important between‐group differences. Hence it is possible that we failed to detect differences in intervention effects that were present. The vast majority of participants were women, thus the results should be generalized to men with caution.

## 5 CONCLUSION

This study showed that the PASS intervention was more successful than IPT for the attainment of self‐selected activity‐related rehabilitation goals with regard to improvements in activity limitations and satisfaction with activity performance as measured by PGPQ. The differences were sustained over time at follow‐ups up to 2 years postintervention. Immediately after completing the intervention, the PASS group reported having done more changes in their life to improve activity performance than the IPT group. The PASS programme emphasized the importance of applying active pain‐ and stress‐coping techniques in personal ‘risk situations’ to manage pain flare‐ups, which appear to support people with persistent tension‐type neck pain to make changes that improve activity performance.

## CONFLICTS OF INTEREST

The authors declare no conflicts of interest.

## AUTHOR CONTRIBUTIONS

Catharina Gustavsson and Lena von Koch have substantially contributed to all parts of the study: the study design, acquisition and analysis of data, interpretation of results and drafting of the manuscript. Both authors have approved the final version of the manuscript and are accountable for all aspects of the work.

## Data Availability

The data that support the findings of this study are available from the corresponding author upon reasonable request.
